# Epidemiological modeling in *StochSS Live*!

**DOI:** 10.1093/bioinformatics/btab061

**Published:** 2021-01-29

**Authors:** Richard Jiang, Bruno Jacob, Matthew Geiger, Sean Matthew, Bryan Rumsey, Prashant Singh, Fredrik Wrede, Tau-Mu Yi, Brian Drawert, Andreas Hellander, Linda Petzold

**Affiliations:** 1 Department of Computer Science, University of California-Santa Barbara, Santa Barbara, CA 93117, USA; 2 Department of Mechanical Engineering, University of California-Santa Barbara, Santa Barbara, CA 93106, USA; 3 National Environmental Modeling and Analysis Center (NEMAC), University of North Carolina at Asheville, Asheville, NC 28804, USA; 4 Department of Information Technology, Uppsala University, Uppsala 751 05, Sweden; 5 Department of Molecular, Cellular and Developmental Biology, University of California-Santa Barbara, Santa Barbara, CA 93106, USA; 6 Department of Computer Science, University of North Carolina at Asheville, Asheville, NC 28804, USA

## Abstract

**Summary:**

We present *StochSS Live!*, a web-based service for modeling, simulation and analysis of a wide range of mathematical, biological and biochemical systems. Using an epidemiological model of COVID-19, we demonstrate the power of *StochSS Live!* to enable researchers to quickly develop a deterministic or a discrete stochastic model, infer its parameters and analyze the results.

**Availability and implementation:**

*StochSS Live!* is freely available at https://live.stochss.org/

**Supplementary information:**

Supplementary data are available at *Bioinformatics* online.

## 1 Introduction

Epidemiological models are essential tools to assist public health authorities in the planning of policy responses to pandemic prevention and control (Thompson, 2020). In general, these models are classified into different categories (deterministic/stochastic), treatment of the populations (continuous/discrete) or spatial dependence and distribution of the population (homogeneous/heterogeneous) (Garner and Hamilton, 2011).

An example of a recent application of epidemiological modeling is the study of early transmission dynamics and effectiveness of control measures in individuals infected by the novel coronavirus disease (COVID-19). As of September 7, 2020, COVID-19 has been responsible for over 27 million reported cases and 900,000 deaths worldwide (World Health Organization, 2020). Given the global impact of the virus, several software tools have been developed, mostly focused on either deterministic (Flaxman *et al.*, 2020) or stochastic (Sanyi *et al.*, 2020) models. These tools typically require some level of technical expertise.

On the mathematical level, most epidemiological models are structurally identical to models of chemical kinetics widely used in systems biology (Hoops *et al.*, 2006; Lopez *et al.*, 2013). In the systems biology community, there has been a large focus the last decade on increasingly efficient stochastic simulation algorithms and on tools to improve usability for modelers. We have in previous work developed a wide range of model development and simulation tools for such models in the *StochSS Suite of Software*. We believe that there is great urgency and potential for a software environment that makes epidemiological modeling easily accessible to a wide audience, and that bridges the notation gap needed to effectively reuse simulation tools from systems biology for epidemiological models. To accomplish this we present *StochSS Live!*, a powerful web-based tool that enables users to create models, perform simulations, infer parameters and visualize the results through simple and intuitive workflows, and have developed a stochastic COVID-19 epidemiological model accessible via *StochSS Live!*

*StochSS Live!* enables easy access to the powerful feature set of the simulation and model analysis toolkits in the *StochSS Suite of Software* (Abel *et al.*, 2016; Singh *et al.*, 2020). *StochSS Live!* builds on and extends the model development UI from (Drawert *et al.*, 2016) in several ways: Through a set of clear, user-friendly interfaces used directly from a web browser (hence requiring no installation), a researcher can explicitly define their model, simulate it using deterministic or stochastic solvers, analyze and explore the parameter space using either traditional parameter sweeps or workflows guided by unsupervised machine learning. Users can also calibrate the model to observed data using highly scalable likelihood-free parameter inference. Specifically, *StochSS Live!* utilizes the Approximate Bayesian Computation (Sisson *et al.*, 2018) algorithms implemented in Sciope (Singh *et al.*, 2020) to provide an easy interface for parameter estimation given a parameterized model and observed data. Other libraries for model calibration such as PyBioFitNet (Mitra *et al.*, 2019), DEAP (Félix-Antoine *et al.*, 2012) or PyDREAM (Shockley *et al.*, 2018) can also be implemented by the user through the Jupyter notebooks generated for each model. For analysis needs that goes beyond the capability of the UI, *StochSS Live!* will automatically generate templated Jupyter notebooks that can be shared and extended. This automated dual representation of models and computational workflows via a UI and as code is a defining feature of *StochSS Live!* and greatly simplifies collaboration between domain and computational experts.

## 2 Epidemiological model

**Fig. 1.**
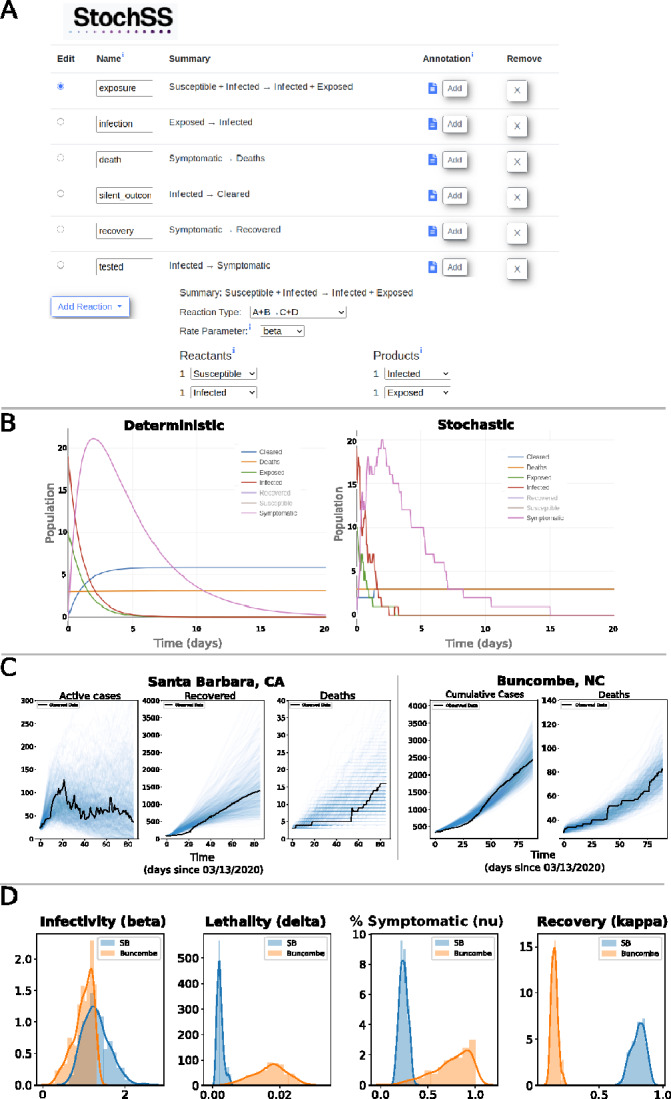
Snapshot of the *StochSS Live!* web interface. (**A**) The user can explicitly define populations, parameters and reactions. (**B**) The preview window settings allow the user to preview simulation results for both deterministic and stochastic models. (**C**) Example of parameter sweep inference in *StochSS Live!* The blue lines are computed realizations obtained by the stochastic solver and the black lines correspond to the official data. Notice that, regardless of the fact that data from Buncombe and Santa Barbara counties have different scales and different levels of stochasticity, *StochSS Live!* is capable of modeling both cases. (**D**) Comparison of posteriors from Santa Barbara and Buncombe counties

To demonstrate the use of *StochSS Live!* for epidemiological modeling, we consider the infection dynamics of COVID-19 in two US counties: Santa Barbara, CA and Buncombe, NC. The data were gathered from Santa Barbara’s Health Department and Dong *et al.* (2020), between March 13 and August 31, 2020. We construct an extended SEIRD model with symptomatic and asymptomatic compartments using the *StochSS Live!* model builder, as shown in Figure 1A. We divide the population into seven groups: susceptible, exposed, infected, symptomatic, recovered, deceased and cleared individuals. Transition events between these groups are shown in Figure 1A. The user can immediately preview sample trajectories from either deterministic or stochastic versions of the system simply by selecting the respective option (Fig. 1B).

Figure 1C and D shows the results of parameter inference for a discrete stochastic version of the model. Inference is performed using Approximate Bayesian Computation, allowing for uncertainty quantification of parameters and predictions. In Figure 1C, each realization (blue lines) corresponds to a simulation using a parameter sample from the posterior distribution, which are contrasted with the data (black lines). Figure 1D shows the posteriors for parameters for both counties. While infectivity rates between the two counties are roughly the same, the estimated lethality rate is a bit higher in Buncombe county, although there is substantial uncertainty. We do note that this particular model does not seem to sufficiently capture the data as evidenced in Figure 1C and would need to be further iterated upon before any strong conclusions can be drawn. For a complete description of the model as well as replicating notebooks, we refer the reader to the Supplementary Information.

## 3 Conclusion

Our model of COVID-19 demonstrates epidemiological capabilities of *StochSS Live!*, a freely available, user-friendly web-based service for the development, simulation and analysis of a wide range of models. To make these capabilities as widely accessible as possible, we provide *StochSS Live!* In addition, the *StochSS Suite of Software* provides the individual tools, if you wish to integrate them into your own software.

## Funding

We acknowledge funding from NIBIB Award 2-R01-EB014877-04A1. No official position or official endorsement should be inferred.

*Conflict of*  *Interest*: none declared.

## Supplementary Material

btab061_Supplementary_Data
