# Supplementary material for: Epidemiological modeling in StochSS Live!
Source: Bioinformatics. 2021 Jan 29;37(17):2787–8. doi: 10.1093/bioinformatics/btab061 (PMC7929247; doi:10.1093/bioinformatics/btab061)
Supplement: btab061_Supplementary_Data [file btab061_supplementary_data.pdf]

Epidemiological Modeling with StochSS Live!

January 2021

This tutorial provides an example of using StochSS Live! to implement a specific epidemiological model and to estimate the parameters for it for a specific county. The same information, along with the replicating code, can be found at https://github.com/StochSS/Covid19_Modeling.

1 Introduction

StochSS Live! is the web interface for developing and investigating stochastic models found at https://live.stochss.org. All results can be replicated by importing this repositories and notebooks into StochSS Live!.

The libraries used by StochSS Live! for simulation and analysis are a part of the StochSS suite of software.

2 Implementing An Epidemiological Model in StochSS Live!

In the following, we describe the epidemiological model we use, and demonstrate how it can be implemented in the StochSS Live! web interface. Then we describe the process of creating a parameter inference workflow for some local COVID19 data.

2.1 Model Description

The epidemiological model we implement is an extended version of the SEIRD model that accounts for symptomatic and asymptomatic cases. The involved compartments (species) are: susceptible (S), exposed (E), infected (I), symptomatic (Y), recovered (R), dead (D), and cleared (C). The system can be visualized as:

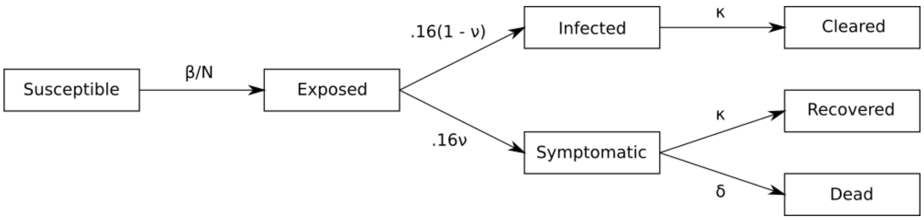

The system evolves according to SEIR dynamics but with a chance of becoming symptomatic after being exposed. We fix the rate at which exposed patients become infectious at 0.16, which represents 6.25 day incubation period and estimate the proportion of patients who become infected vs. symptomatic. This is roughly adopted from a similar model [3]. Specifically, we have the following set of reactions:

$$
\begin{aligned}
\text{Susceptible} + \text{Infected} &\rightarrow \text{Infected} + \text{Exposed} \\
\text{Exposed} &\rightarrow \text{Infected} \\
\text{Exposed} &\rightarrow \text{Symptomatic} \\
\text{Symptomatic} &\rightarrow \text{Recovered} \\
\text{Symptomatic} &\rightarrow \text{Dead} \\
\text{Infected} &\rightarrow \text{Cleared}
\end{aligned}
$$

This model assumes that *only asymptomatic transmission is possible*, *all asymptomatic cases recover*, and that *all parameters are static*.

2.2 Implementation

Using this specification, we can implement the model in StochSS Live! in the model creation interface

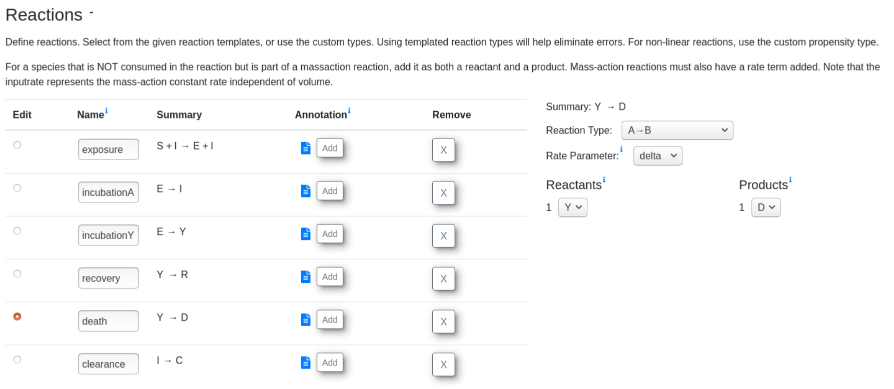

A pre-implemented version of this model with some default parameters can be found here.

In the model creation interface, we can also preview trajectories if we were to consider the model as either discrete stochastic or an ODE model.

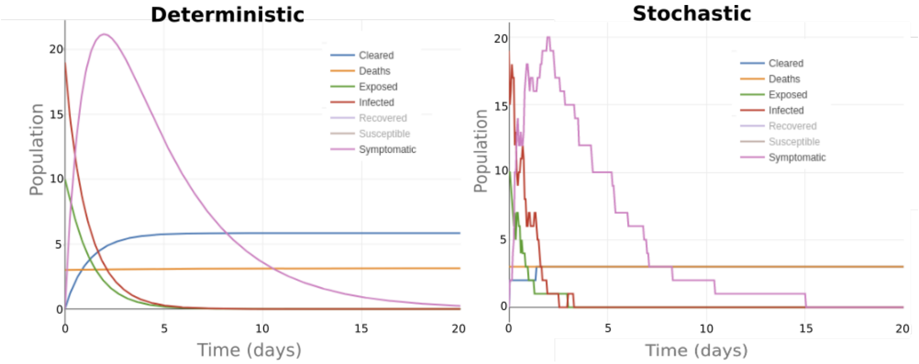

3 Parameter Estimation Worflow using ABC

We estimate the parameters of the model for Santa Barbara and Buncombe counties using the Sciope [4] toolbox, part of the StochSS suite of software which offers model exploration and parameter estimation. A pre-implemented template notebook can be generated by using the "Sciope Model Inference" workflow in StochSS Live!.

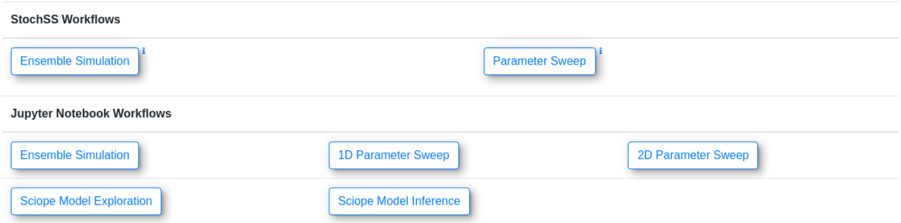

The completed workflow is included for Santa Barbara, CA and for Buncombe, NC.

3.1 Reading In Data

Data for estimating parameters should be loaded in the data block. The `obs_data` object should contain the final completed dataset.

Data

In [3]:
```
import pandas as pd
import datetime

df = pd.read_csv("../data/SBC_Covid19_cleaned.csv")
df = df[pd.to_datetime(df['Date']) > datetime.datetime(2020, 6, 1)]
sb_cases = df[df['Area'] == "CITY OF SANTA BARBARA and the unincorporated area of Mission Canyon"]
goleta_cases = df[df['Area'] == "CITY OF GOLETA"]
iv_cases = df[df['Area'] == "COMMUNITY OF ISLA VISTA"]

confirmed = sb_cases['Active'].values + goleta_cases['Active'].values + iv_cases['Active'].values
recovered = sb_cases['Recovered'].values + goleta_cases['Recovered'].values + iv_cases['Recovered'].values
dead = sb_cases['Deaths'].values + goleta_cases['Deaths'].values + iv_cases['Deaths'].values
obs_times = (pd.to_datetime(sb_cases['Date']) - pd.to_datetime(sb_cases['Date'].iloc[0])).dt.days.values # observat

confirmed_interp = np.interp(np.linspace(0,obs_times[-1],obs_times[-1] + 1), obs_times, confirmed)[5:]
recovered_interp = np.interp(np.linspace(0,obs_times[-1],obs_times[-1] + 1), obs_times, recovered)[5:]
dead_interp = np.interp(np.linspace(0,obs_times[-1],obs_times[-1] + 1), obs_times, dead)[5:]

# obs_data should contain the observed dataset
obs_data = np.vstack([confirmed_interp, recovered_interp, dead_interp]).reshape(1,3,-1)
```

3.2 ABC Requirements

Sciope implements various algorithms for Approximate Bayesian Computation [5]. To use these, we need to complete the following segments of the notebook:

1. Prior cell

Prior Distributions

In [4]:
```
parameter_names = ['beta', 'kappa', 'delta', 'nu']
lower_bounds =    [0     ,       0,       0,     0]
upper_bounds =    [3     ,       1,     0.1,     1]
prior = UniformPrior(np.array(lower_bounds), np.array(upper_bounds))
```

2. Simulator function This function should take in a parameter array and output a simulation from the model that matches the shape of the observed data.

Simulator

In [5]:
```
# Here we use the GillesPy2 Solver
def simulator(params, model):
    res = model.run(
            solver = compiled_solver,
            show_labels = True,
            seed = np.random.randint(1e8),
            variables = {parameter_names[i] : params[i] for i in range(len(parameter_names))})

    # Extract only observed species
    symptomatic = res['Y']
    recovered = res['R']
    dead = res['D']

    return np.vstack([symptomatic, recovered, dead])[np.newaxis,:,:]

# Wrapper, simulator function to abc should should only take one argument (the parameter point)
def simulator2(x):
    return simulator(x, model=model)
```

3. Summary Statistics and Distance Functions

Summary Statistics and Distance Function

In [6]:
```
from sciope.utilities.summarystats.identity import Identity
from sciope.utilities.distancefunctions.euclidean import EuclideanDistance

normalization_values = np.max(obs_data, axis = 2)[0,:]
def max_normalization(data):
    dc = data.copy().astype(np.float32)
    dc[0,0,:] = dc[0,0,:]/normalization_values[0]
    dc[0,1,:] = dc[0,1,:]/normalization_values[1]
    dc[0,2,:] = dc[0,2,:]/normalization_values[2]
    return dc

summary_stat = Identity(max_normalization)
distance_func = EuclideanDistance()
```

3.3 Estimating Parameters and Analyzing Posteriors

The default algorithm we use is Replenishment ABC-SMC [2]. Sciope uses Dask [1] to parallelize inference so we use the StochSS Live! servers to use more processes.

Inference

In [8]:
```
# Start abc instance
from sciope.inference.rep_smc_abc import ReplenishmentSMCABC

abc = ReplenishmentSMCABC(obs_data, # Observed Dataset
                          lambda x : (simulator2(x), 1), # Simulator method
                          prior,
                          summaries_function=summary_stat.compute,
                          ) # Prior
```

In [9]:
```
import dask
with dask.config.set(scheduler = 'processes', workers = 20):
    smc_abc_results = abc.infer(num_samples = 1000)

posterior = smc_abc_results['accepted_samples']
```

The inference returns a `np.array` of samples from the the posterior distribution stored in the posterior object. Each sample can be used as a set of parameters in the model to generate further trajectories.

Below, we show the posterior distribution of parameters for Santa Barbara as well as generated data from the model using the posterior samples (posterior predictive).

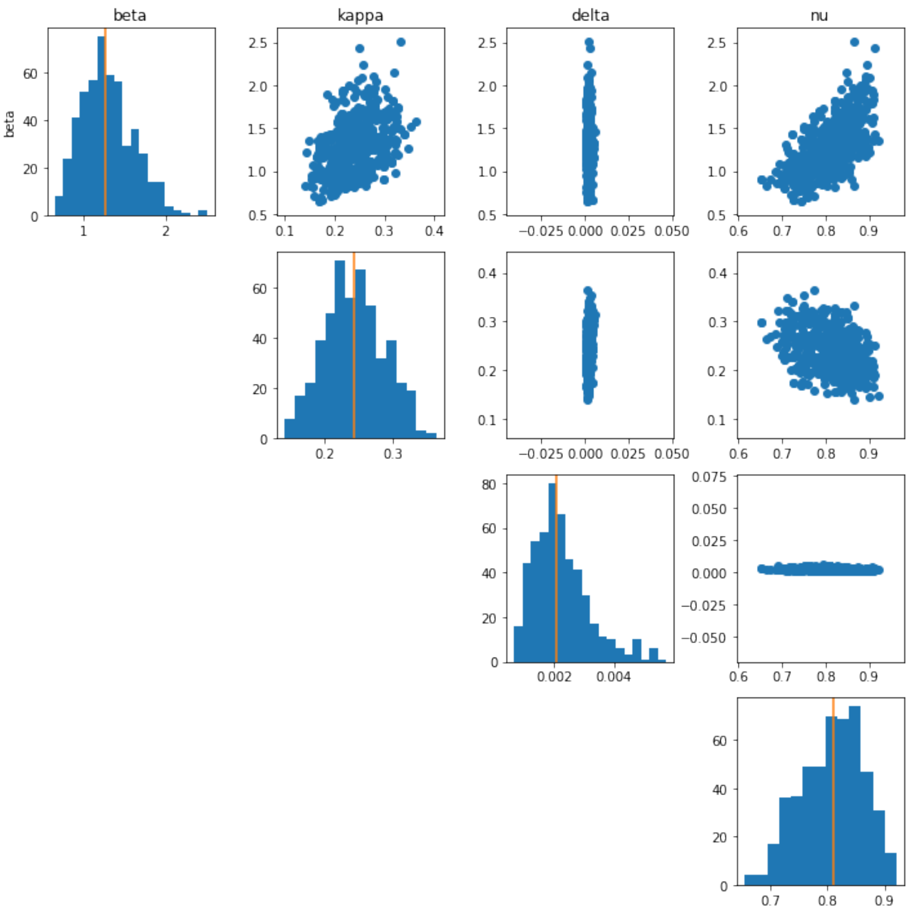

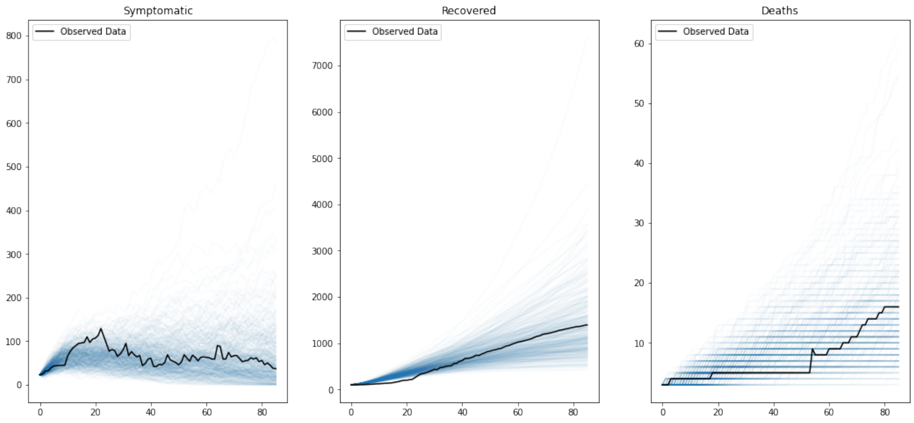

We note that the presented models do not really indicate a sufficient fit to

the data to draw any strong conclusions. For a complete analysis, this process needs to be repeated, changing the model to better capture assumptions about the system. For example, we would expect the infectivity to change over time as policies are implemented and we know that there are non-intrinsic measurement error, such as reporting errors in the data.

References

[1] Dask Development Team. Dask: Library for dynamic task scheduling. 2016.

[2] C. C. Drovandi and A. N. Pettitt. Estimation of parameters for macroparasite population evolution using approximate bayesian computation. *Biometrics*, 67(1):225–233, 2011.

[3] S. Flaxman, S. Mishra, A. Gandy, H. J. T. Unwin, T. A. Mellan, H. Coupland, C. Whittaker, H. Zhu, T. Berah, J. W. Eaton, et al. Estimating the effects of non-pharmaceutical interventions on covid-19 in europe. *Nature*, 584(7820):257–261, 2020.

[4] P. Singh, F. Wrede, and A. Hellander. Scalable machine learning-assisted model exploration and inference using Sciope. *Bioinformatics*, 07 2020. btaa673.

[5] S. A. Sisson, Y. Fan, and M. Beaumont. *Handbook of approximate Bayesian computation*. CRC Press, 2018.
